# Effectiveness of preoperative bath using chloroxylenol antiseptic soap on the incidence of post emergency cesarean section surgical site infection at Mbarara Regional Referral hospital, Uganda: a randomized controlled trial

**DOI:** 10.11604/pamj.2022.41.92.23687

**Published:** 2022-02-02

**Authors:** Henry Lukabwe, Rodgers Kajabwangu, Dale Mugisha, Horace Mayengo, Baraka Munyanderu, Asanairi Baluku, Anthony Manyang, Jolly Joe Lapat, Francis Banya, Musa Kayondo, Ronald Mayanja, Joy Muhumuza, Francis Bajunirwe, Joseph Ngonzi

**Affiliations:** 1 Mbarara University of Science and Technology, Department of Obstetrics and Gynecology, Mbarara, Uganda,; 2 Kisiizi Church of Uganda Hospital, Kabale, Uganda,; 3 Mbarara University of Science and Technology, Department of Community Health, Mbarara, Uganda

**Keywords:** Incidence, surgical site infection, chloroxylenol, Mbarara University, Uganda

## Abstract

**Introduction:**

Surgical Site Infections (SSIs) constitute 15%-45% of hospital acquired infections in sub-Saharan Africa. Cesarean section (CS) increases the risk of developing sepsis by 5-20 times and is highest when the operation is emergency. Therefore, the purpose of this study was to measure the effect of chloroxylenol in reducing the incidence of post cesarean SSIs at Mbarara Regional Referral Hospital (MRRH).

**Methods:**

a randomized controlled trial was conducted at MRRH maternity ward and mothers were randomized into either control or intervention arms. The intervention was a complete body bath with chloroxylenol antiseptic soap before the operation, while the control arm participants received a standard ward pre-operative preparation procedures. All participants were followed up for 30 days and assessed using an SSI screening tool.

**Results:**

ninety-six women were randomized, and 48 were assigned to each arm. The overall incidence of SSI was 30.21%. The incidence of SSI was significantly lower in the intervention compared to the control arm (6.25% in the intervention arm versus 54.17% in the control arm) (p-value <0.001). Chloroxylenol bath was protective of SSI with a 90% risk reduction for SSI (95% confidence interval of 67%–97%).

**Conclusion:**

a preoperative bath with chloroxylenol for pregnant mothers is associated with a significantly lower risk of post cesarean section surgical site infections. Health facilities with a high burden of post SSI should consider adding this simple and effective intervention to the existing infection prevention measures. Clinical Trials.gov registration (NCT03544710).

## Introduction

Surgical site infections (SSI) are infections that occur at a wound following an invasive surgical procedure. Surgical site infections are infections related to the surgical procedure and occur at or near the surgical incision within 30 days of the surgery [1]. Surgical site infections are the commonest hospital acquired infections in sub-Saharan Africa [2], estimated at 15%-45% [3-5]. The incidence of SSIs in the middle and high income settings is much lower and ranges between 1.8-5.5% [5].

Cesarean delivery is the single-most important risk factor for postpartum pregnancy-associated infections carrying a 5 to 20-fold increase in the risk of developing sepsis [6]. Puerperal sepsis, which is part of the SSI broad spectrum, is the leading cause of maternal mortality at Mbarara Regional Referral Hospital (MRRH) [7]. Emergency Cesarean sections have been associated with a higher risk of infections than elective Cesarean sections [8]. Despite several interventions to reduce postpartum infections after Cesarean section the burden of SSI remains high [9]. There is a need for cost effective interventions with potential to scale widely, to tackle the challenge of SSI in sub-Saharan Africa and other resource limited settings. One such intervention is an antiseptic bath. Chloroxylenol, also known as para-chloro-meta-xylenol, is an antiseptic and disinfectant used for disinfecting skin and surgical instruments. It was first made in 1927 and is on the World Health Organization´s Essential Medicines list. It is a common constituent of household products like medicated soap, and antiseptics [10]. Chloroxylenol works by disrupting the cell wall and by stopping enzyme function [11].

Preoperative antiseptic bathing reduces skin microbial colony counts and hence risk of wound contamination. There is no conclusive evidence whether the practice has a protective effect on the incidence of SSIs in a surgical setting [4]. Preoperative bathing of patients is currently not practiced routinely in sub-Saharan Africa and therefore its impact on reducing the burden of SSI is unknown. Therefore, the purpose of this study was to assess whether an antiseptic soap bath of chloroxylenol is associated with reduction in the incidence of surgical site infections among post Cesarean women at a large urban hospital in a resource limited setting.

## Methods

**Study site and setting:** we conducted a randomized controlled single blind trial between November 2017 and February 2018 precisely at the antenatal, labor and postnatal units of Mbarara Regional Referral Hospital (MRRH), in southwestern Uganda.

**Current practice:** all mothers scheduled for cesarean section had an intravenous cannula inserted and were administered ampicillin 2g intravenously for prophylaxis. The women had a blood sample of 3mls drawn for blood grouping and cross matching to secure a unit of blood in case they needed a transfusion. Mothers then received 1 liter of intravenous normal saline, and had a urethral catheter placed for drainage of urine and written consent was obtained from them. Theatre was notified to prepare for the operation. There was no mandatory operative bath. Following delivery, and regardless of the mode of delivery, mothers are followed-up by a physician in charge of treatment adjustment in case of need, pain management and post-partum infection prevention or treatment using ceftriaxone, metronidazole and ampicillin.

**Inclusion criteria:** all women delivering by emergency cesarean section at MRRH who accepted to give informed consent.

**Exclusion criteria:** obvious evidence of infection such as fever, foul-smelling liquor, or those already on antibiotics for reasons other than preoperative prophylaxis. Women in whom emergency caesarian delivery was expected to occur within less than 30 minutes such as those with fetal distress, obstructed labor, pulsatile cord prolapse, or ruptured uterus. Women who could not communicate and give information for the study and those who did not have access to an active cell phone contact for purposes of follow up were also excluded.

**Participant enrollment and randomization:** all women who met the selection criteria were consecutively enrolled into the study by the mid wife research assistant, who was not part of the clinical team and study participants were enrolled until the sample size was attained. The research assistant obtained written informed consent from each study participant and collected demographic and baseline clinical information. Informed consent for the surgery was obtained separately from that for the research. The study participants were then randomly assigned to either the intervention or the control by picking a sealed envelope from the randomization basket. The envelopes contained the label of the study arms. The group of assignment was determined when the envelope was opened.

**Intervention:** women allocated to the intervention arm received a tablet of soap containing chloroxylenol antiseptic and they were asked to use the soap to take a full body bath. The research assistant provided warm water to the participants and she further supervised the bathing process to ensure that the entire body, apart from the hair, had been washed. The bathing process on average lasted twenty minutes. Following the bath, the participants were dressed in a clean theatre gown, and received the care described above as 'current practice'.

**Control arm:** participants randomized to the control group received the routine pre-operative procedures provided to all emergency caesarian section patients as a current practice. The routine pre-operative care included establishing an intravenous cannula, intravenous normal saline fluids for preloading, intravenous pre-operative antibiotics (ampicillin), informed written consent, blood for grouping and cross matching plus informing theatre staff. If a participant in the control arm requested to have a bath before the operation, they were provided with warm water and a non-medicated soap for their bath. Participants who had a bath with non-medicated soap were still eligible to remain in the control arm.

**Follow-up procedures:** the research assistant and the ward clinical team in charge of assessment of the patients for clinical outcomes were blinded to the arm of the patients. The patient assessments were conducted daily until the date of discharge. The case report forms were completed by the research assistants using information from the clinical notes in the patients´ charts. Information on the clinical evidence of SSIs was obtained by the clinical team and written in the patients´ charts. Participants were asked whether they had severe pain at the incision site and pain was regarded as severe if the participant reported that it stopped her from moving out of bed. Axillary body temperature, radial pulse rate and respiratory rate were measured twice daily. The incision site was examined for induration (edema or erythema), purulent discharge, and severe tenderness as evidenced by guarding. The temperature of the skin around the incision site was compared with that of the skin remote from the incision site to determine whether it was warmer around the incision site. The vaginal pad was examined in order to assess the nature of the per-vaginal discharge.

The decision to discharge the participants from hospital was made by the ward clinical team. Upon discharge from the hospital, the participant was given an appointment to return on the 7^th^ post-operative day for follow up. At this visit, they were asked questions and examined, all following the same procedures as those during their admission to evaluate for evidence of SSI. If a participant failed to turn-up for the appointment, they were contacted on phone by a member of the research team and requested to return and for those who were not able, the research assistant would locate their residence and travel there for the follow up. At the 30^th^ day post-operative, the research assistant followed up the participants through a telephone call and asked them questions about how their health had been from their last visit (7^th^ post-operative day) to the current date. Participants were asked questions about feeling feverish, severe lower abdominal pain, or any discharge from the incision site, or a foul-smelling per-vaginal discharge, or if they had sought medical care from a facility due to issues related to operation site since their last hospital visit. Participants had been given a hotline to call a member of the research team in case they had a medical problem. All participants that met the criteria of SSI at any time were referred to the existing clinical care system in the department of Obstetrics and Gynecology at Mbarara Regional Referral Hospital for treatment.

**Measurements:** socio-demographic data were collected including age in years, parity, address or village of residence, marital status, educational level, occupation, income, and partner's factors including income, occupation, and education level. We collected information on obstetric care factors like indication for the cesarean section, stage of labor, status of membranes, number of digital vaginal examinations done, prophylactic antibiotics (whether given or not, which one given, and how long before the incision was it given), the training level of the primary surgeon and assistant surgeon, day and time of incision, length of the operation, skin closure technique, suture material used for closure, a completely filled WHO surgical checklist. We also collected data on history of medical conditions like diabetes mellitus, sexually transmitted diseases, HIV, and urinary tract infections.

**Primary study endpoint:** the primary outcome was surgical site infection following caesarean section delivery, defined as infection involving skin, subcutaneous tissue, fascial layer, muscle or organs, occurring with at least one of the following; 1) purulent discharge from the incision site; 2) spontaneous wound dehiscence; 3) surgeon deliberately opens the wound when the patient has at least one of the following; pain or tenderness, localized swelling or induration, redness, or heat; 4) an abscess or evidence of infection involving the deep incision or organ space found on direct examination, during reoperation, or by ultrasonography; 5) diagnosis of surgical site infection by attending surgeon.

**Sample size calculation:** we conducted sample size calculations for randomized controlled trials using the Kelsey *et al*. formula [12]. We estimated the incidence of SSI to be 26% [3] and assumed that administration of the pre-CS bath would result in a 75% reduction in incidence of SSI. A sample size of 48 participants per arm would detect such a difference or more with 80% power, using a 5% level of significance.

**Data management and analysis:** we examined the completed case report forms, screening tools and phone call interview sheets on each day for completeness before storage in a lockable cabinet. The data were then entered into a Research Electronic Data Capture (REDCap) database [13] and later exported to STATA 13.0 software for cleaning and analysis. In the analysis, baseline characteristics were stratified by intervention arm and summarized using proportions for categorical variables and Chi-square tests were performed to compare proportions. Fisher's exact test was used in case the expected numbers in the cells was less than 5. Continuous variables were categorized and analyzed as other categorical variables. The statistical tests were performed at a level of significance of 0.05. Our aim was to present the intention to treat analysis as the primary analysis. We compared the incidence of SSI in the intervention and control group, and calculated the relative risk (RR) for SSI in the intervention and control arms. We calculated the risk reduction for SSI in the intervention as 1-RR and computed the 95% confidence interval.

**Ethics approval:** the study and enrollment procedures were approved by the Mbarara University of Science and Technology Research ethics committee (08/08-17), the trial was registered at the portal Clinical Trials.gov (NCT03544710) and received the final approval from the Uganda National Council of Science and Technology (HS/215ES).

## Results

**Figure 1:**
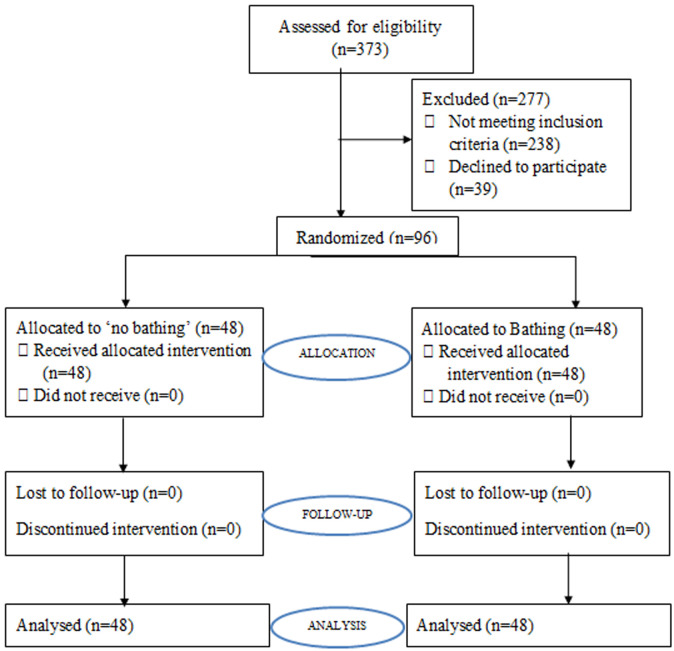
consort diagram showing the recruitment process

**Participants recruitment process:** as shown in Figure 1, we screened 373 patients and 277 participants were excluded due to various reasons as follows: 113 participants had indications for cesarean section that necessitated the operation to be done within 30 minutes, 41 participants were already on antibiotics, while 124 participants did not have access to a working telephone, and 39 participants declined taking part in the study. All participants recruited were included in the final analysis and no participant was lost to follow-up, nor withdraws after recruitment.

**Socio-demographics of study participants:** there was a uniform distribution for the majority of the participants' socio-demographics among the two study groups. Over 60% of the participants reported first degree relatives as their primary care takers (62.5% in the intervention and 755 in the control arm) as shown in Table 1.

**Table 1:** socio-demographics of study participants, comparing those who received intervention and those in the control group

Characteristic (n=96)	Intervention n (%), (n=48)	Control n (%), (n=48)	P-value*
**Age category**			0.306
<20	9 (18.8)	11 (22.9)	
20-24	19 (39.6)	13 (27.1)	
25-29	11 (22.9)	18 (37.5)	
30-37	9 (18.8)	6 (12.5)	
Address; Rural	34 (70.8)	24 (50.0)	0.037
Married	47 (97.9)	48 (100)	0.315
**Education level**			0.492
None	6 (12.5)	2 (4.2)	
Primary	21 (43.8)	21 (43.8)	
O'level	15 (31.3)	17 (35.4)	
Tertiary	6 (12.5)	8 (16.7)	
**Occupation**			0.641
Peasant farmer	17 (35.4)	20 (41.7)	
Business	16 (33.3)	17 (35.4)	
Professional	15 (31.3)	11 (22.9)	
Income <100000	29 (60.4)	22 (45.8)	0.152
**Partners factors**			0.797
**Education level**			
None	3 (6.4)	3 (6.3)	
Primary	19 (40.4)	17 (35.4)	
O'level	18 (38.3)	17 (35.4)	
Tertiary	7 (14.9)	11 (22.9)	
Occupation			0.343
Peasant farmer	12 (25.5)	7 (14.6)	
Business	30 (63.8)	33 (68.8)	
Professional	5 (10.6)	8 (16.7)	
Income			0.331
<100000UGX	9 (19.2)	5 (10.4)	
100000-299999.99UGX	24 (51.1)	23 (47.9)	
≥300000UGX	14 (29.8)	20 (41.7)	
Supportive partner	46 (97.9)	47 (97.9)	0.988
Primary caretaker			
First degree relative	30 (62.5)	36 (75.0)	0.310
Other relatives	13 (27.1)	7 (14.6)	
Friends/ none	5 (10.4)	5 (10.4)	

O' level-ordinary secondary school education level; UGX-Uganda shillings

**Obstetric care factors and medical factors of study participants:** participants did not differ in their obstetric care factors across the two study groups. A higher number of participants with primary caesarean section was in the intervention arm (85.4%) compared to 62.5% in the control arm. More participants in the control arm had 4 or more vaginal examinations (70.8%) compared to 43.8% in the intervention arm. Majority of the participants were in active stage of labor. Junior residents were the leading primary surgeons whereas intern doctors assisted in most of the operations. Majority of the participants had their skin closed with the non-absorbable suture, as shown in Table 2.

**Table 2:** obstetric care factors and medical factors of study participants, comparing those who bathed (intervention group) and those who did not bathe (control group)

Characteristic (n=96)	Intervention n (%), (n=48)	Control n (%), (n=48)	P-value*
**Obstetric factors**			
Primi-parity	29 (60.4)	25 (52.1)	0.411
Primary cesarean	41 (85.4)	24 (62.5)	0.011
**Stage of labor at cesarean**			
Latent phase of first stage	5 (10.4)	9 (18.8)	0.208
Active phase of first stage	37 (77.1)	37 (77.1)	
Second stage	6 (12.5)	2 (4.2)	
Ruptured membranes before cesarean	35 (72.9)	33 (68.8)	0.653
Vaginal examinations done ≥4	21 (43.8)	34 (70.8)	0.007
Indication of cesarean			0.519
Prolonged labor	21 (43.7)	17 (35.4)	
Mal-presentation	20 (41.7)	20 (41.7)	
Others	7 (14.6)	11 (22.9)	
Not given prophylactic antibiotics	5 (10.4)	3 (6.3)	0.460
Operation done over weekend	12 (25.0)	33 (29.2)	0.646
Operation done in the night	23 (47.9)	23 (47.9)	1.000
Operation lasting ≥60	6 (12.5)	11 (22.9)	0.181
Cadre of primary surgeon			0.193
Senior resident	20 (41.7)	12 (25.0)	
Junior resident	26 (54.1)	32 (66.7)	
Intern doctor	2 (4.2)	4 (8.3)	
Cadre of assistant surgeon			0.370
Senior resident	3 (6.3)	4 (8.3)	
Junior resident	4 (8.3)	1 (2.1)	
Intern doctor	41 (85.4)	43 (89.6)	
Subcutaneous skin closure technique	14 (29.2)	21 (43.8)	0.138
Absorbable suture material used	10 (20.8)	17 (35.4)	
**Medical factors**			0.112
Urinary tract infections	24 (50.0)	38 (79.2)	0.003
HIV-infection	1 (2.1)	4 (8.3)	0.168
Sexually transmitted diseases	23 (47.9)	17 (35.4)	0.214

C/S: cesarean section; VEs: digital vaginal examinations; HIV: human immunodeficiency virus

**Incidence of surgical site infection in the study groups:** Table 3 shows that the incidence rate of SSI was lower among mothers of the intervention group compared to the control group (6.25 and 54.17 respectively). Thus, preoperative bathing with chloroxylenol gives an absolute risk reduction or risk difference of getting SSI of 47.92%, and a relative risk reduction of 88.5%.

**Table 3:** incidence of SSI in the intervention and control groups

	Control, n (%)	Intervention, n (%)	Total, n (%)
No SSI, n (%)	22 (45.8)	45 (93.7)	67 (69.8)
SSI, n (%)	26 (54.2)	3 (6.3)	29 (30.2)
Total, n (%)	48 (100)	48 (100)	96 (100)

SSI: surgical site infection)

**Obstetric care factors associated with post cesarean section surgical site infection among all study participants:** in Table 4, preoperative bathing was found to be the only significant obstetric care factor with an unadjusted risk ratio of 0.1 at a 95% confidence interval of [0.04-0.36] and a level of significance of p-value <0.001. No medical factor was found to be significant for surgical site infection. After adjusting for number of vaginal examinations done, pre-operative baths were associated with a 90% reduction in incidence of SSI (95% CI 67%-97%).

**Table 4:** multivariate logistic regression analysis of obstetric care factors associated with post cesarean section surgical site infection among all study participants

Characteristic	Unadjusted		Adjusted	
(n=96)	RR (95% CI)	P-value*	RR (95% CI)	P-value*
Pre-operative bathing	0.1 [0.04-0.36]	<0.001	0.1 [0.03-0.33]	<0.001
Number of VEs done	0.9 [0.50-1.69]	0.782	1.4 [0.83-2.22]	0.224

VEs: vaginal examinations

**Adverse events:** we did not receive complaints of allergic reactions, irritations or record any dermatitis or other adverse effects associated with the use of the soap.

## Discussion

The study found out that pre-operative antiseptic bathing using chloroxylenol containing soap reduced the incidence of post cesarean section surgical site infection at Mbarara Regional Referral Hospital with a risk reduction of more than 80% in the incidence of SSI. The study found out that the overall incidence of SSI among post cesarean mothers at our hospital was very high, at over 50% in the control group. The evidence provides a strong tool to potentially reduce SSI in similar settings in sub-Saharan Africa, where there is a high volume of cesarean delivery with a high incidence of SSI, such as Uganda.

Our study therefore shows that pre-operative bath among mothers undergoing caesarean section is effective in reducing SSI in a busy maternity ward. To the best of our knowledge, this is the first study to evaluate the effectiveness of this simple and inexpensive intervention on SSI in sub-Saharan Africa. We found a study that examined the effect of pre-operative baths on SSI [14], and found there was a significant benefit in the intervention. However, this study was significantly different from our study in several ways. First, this study was conducted among a general surgical population. Second, the study was a multi-modal approach with several interventions embedded in the design in addition to the pre-operative baths. These fundamental differences make it impossible to make any comparisons between the two studies. However, both studies share the common finding that pre-operative baths can significantly reduce SSI.

The burden of postpartum infections at our study site and many settings in sub-Saharan Africa is very variable but mostly high [3,4,15,16]. Previous studies have shown that mothers who undergo emergency cesarean sections are at a higher risk for SSI compared to those undergoing elective procedures [17]. Mothers that need emergency CS are usually referred from peripheral rural sites, have travelled long distances, not prepared for surgery, with some mothers involved in farming, brought to the hospital straight from their gardens, exposing them to high risk for infections. These infections contribute significantly to adverse maternal outcomes [18]. Data from one of our previous studies and many others have shown that puerperal sepsis is the leading cause of maternal mortality in the hospital [7,19]. Finding such a high incidence of SSI among post emergency Cesarean section mothers only confirms the burden as being huge, and one that needs extra attention.

Research conducted elsewhere shows incidence rates for SSI lower than what we found in our study. Jimma University Teaching Hospital, Ethiopia, whose setting is very similar to that of Mbarara Regional Referral Hospital, found the post Cesarean section SSI rate to be 11.4% [20]. The study found a lower incidence probably because they followed up all post cesarean section mothers, including elective cesarean sections, who are at a lower risk for SSI. Similarly, studies done in Bugando Medical Centre, Mwanza, and in Kiambu District hospital in Kenya had lower incidence rates of SSIs of 10.9% and 19% respectively probably because both studies enrolled elective and emergency cesarean section mothers [21,22]. Our study enrolled only emergency Cesarean section mothers. It is well known that emergency cesarean sections have higher rates of SSI than elective Cesarean sections.

Chloroxylenol is an inexpensive antiseptic that is in common use in hospital settings and outside hospital setting in household items such as antibacterial soaps. It is an effective component of antibacterial soaps and has demonstrated a significant reduction in fecal coliforms in hand-washing experiments [23]. Given its antibacterial properties, there are concerns about potential contribution to the emergence of bacterial resistance if the use is widespread, especially outside clinical settings. And the widespread use is likely to grow following a ban on triclosan and triclocarban in antibacterial soaps by the Food and Drug Administration of the United States [24]. Antibacterial soaps are not widely used in households in sub-Saharan Africa, and therefore the potential emergence of bacterial resistance is unlikely to be a challenge in foreseeable future. Also of concern is the possible dermatitis associated with antibacterial soaps. Certain fragrances in these soaps are associated with contact dermatitis [25].

In our study, we did not record any dermatitis associated with the use of the soap possibly due to the small concentration of chloroxylenol in the soap used. However, we recommend that such adverse effects should be evaluated if this intervention is to be implemented.

Our study had some potential limitations. First, we were not able to have all the participants return to the clinic to have an objective clinical assessment. Instead, we followed them up via phone and therefore relied on the self-reports. Second, we did not culture the micro-organisms to identify the cause of the SSI. However, other studies in the region have been done and the common organisms that cause SSI and the drug sensitivity pattern have been studied and are now known. It's also possible that the non-medicated soap used by women in the control arm who requested for a bath before the operation could have had some effect on the micro-organisms. This was however not measured. Nevertheless, being a randomized controlled trial, and the ability to complete follow-up of all participants to 30 days with no loss to follow-up make this study strong. Secondly, we evaluated a simple and inexpensive intervention that is effective, easy to implement and therefore has potential for wide scale impact. And lastly, to the best of our knowledge, this is the first study of its kind to evaluate this intervention in a randomized controlled study among caesarean mothers. A recent multi-centric study that examined the impact of pre-operative baths, was done among a general surgical population and in addition, the baths were not with chloroxylenol, and also this study had a multi-modal intervention approach with a before-after design, and not a randomized controlled design.

## Conclusion

Pre-operative bathing with chloroxylenol soap reduces the risk of post Cesarean section surgical site infections. In a tertiary hospital which has a high burden of postpartum infections, adding this cheap, yet efficacious practice on the already existing infection prevention measures will contribute to reduction on the burden.

**Availability of data and materials:** the datasets used and/or analyzed during the current study are available from the corresponding author on reasonable request.

**Consent for publication:** written informed consent for publication was obtained from the participants.

**Funding:** this research was largely (about 90%) funded by Church of Uganda Kisiizi Hospital, Uganda. The funding organization had no role in the collection, analysis and or interpretation of the data.

### What is known about this topic

- Cesarean section is a risk for postpartum sepsis;
- Postpartum sepsis can cause maternal death;
- Emergency Cesarean sections carry a higher risk of infection than electives.

### What this study adds

- The incidence of SSI is 30.21%;
- Chloroxylenol bath is protective of SSIs;
- Implementation of pre-operative births is feasible in a busy maternity ward.
